# Dynamics of the stream of thought: insights from an accumulation-to-threshold model

**DOI:** 10.1093/nc/niag045

**Published:** 2026-08-14

**Authors:** Adrien Kérébel, Jérôme Sackur

**Affiliations:** Laboratoire de Sciences Cognitives et Psycholinguistique (LSCP), Département d’Études Cognitives de l’École Normale Supérieure (ENS), Centre National de la Recherche Scientifique (CNRS), Paris Sciences et Lettres (PSL) Research University, 29 rue d’Ulm, 75005 Paris, France; Laboratoire de Sciences Cognitives et Psycholinguistique (LSCP), Département d’Études Cognitives de l’École Normale Supérieure (ENS), Centre National de la Recherche Scientifique (CNRS), Paris Sciences et Lettres (PSL) Research University, 29 rue d’Ulm, 75005 Paris, France; Ecole des Hautes Etudes en Sciences Sociales (EHESS), 54 Bd Raspail, 75006 Paris, France

**Keywords:** stream of thought, spontaneous thought, attention, accumulation models, metacognition, ADHD

## Abstract

The dynamics of the stream of spontaneous thoughts has been the object of increasing research in recent years. Here, we study the timing of transitions from one coherent thought episode to the next as one aspect of such dynamics. Participants either vocalized freely generated words or allowed their minds to wander during 2- to 3-min runs, and were instructed to report thought transitions by pressing a key. We first validate this subjective mental event segmentation, which enables us to measure the duration of coherent thought episodes. We then turn to model comparison to show that the timing of thought transitions is best explained by an accumulation-to-threshold mechanism. We further find that the threshold of this accumulation negatively correlates with participants’ trait tendency towards disorganized thought. We speculate about the nature of the accumulated information and emphasize that individual variability in transition thresholds underscores the role of mental impulsivity in thought transitions.

## Introduction

The unfolding of conscious thought in time is still an enigma in consciousness research. In modern psychological science, its investigation can be traced back to James’ famous description of the “stream of thought” (James 1890). He emphasized its dynamic aspect: thought content evolves in time at a variable pace, alternating between stable and unstable phases. This aspect is particularly salient in spontaneous thought, i.e. thought processes that arise in non-demanding contexts. Most investigations of this type of cognition have focused on one of its principal forms, namely mind-wandering, commonly defined as task-unrelated or stimulus-independent thought (Smallwood and Schooler 2015). To our knowledge, there has been no systematic attempt to quantify the overall prevalence of spontaneous thought episodes in general, but the subset of mind-wandering has been estimated to occupy between 30% and 50% of our waking hours (Kane et al. 2007, Killingsworth and Gilbert 2010). This suggests that spontaneous thought, more broadly, is at least as prevalent, making it an important object of study. In addition, abnormal spontaneous thought patterns seem to be at the core of some psychiatric conditions (e.g. racing thought in Attention deficit hyperactivity disorder, ADHD), some of which actually extend as a continuum in the general population (Larsson et al. 2012, Asherson and Trzaskowski 2015, McLennan 2016).

In the last few years, methodological advances led to the development of quantitative investigations of spontaneous thought, aiming at characterizing its dynamics. Experimental protocols typically require participants to report the unfolding of their thought in time, either in a free format, or using more constrained word association tasks (Sripada and Taxali 2020, Andrews-Hanna et al. 2021). Two broad findings have already emerged using such protocols. First, the stream of thought consists of a succession of coherent thought segments, punctuated by various kinds of transitions. Sripada and Taxali (2020) argued for a “clump-and-jump” structure of the stream of thought, where thoughts about a similar topic cluster and are delimited by abrupt transitions to other topics, and Kérébel et al. (2024) found evidence for the existence of a buffer period of unfocused thinking in some transitions. These findings reinforce earlier research about the structure of the stream of thought (e.g. Klinger 1978). Second, the dynamics of spontaneous thought vary across individuals in the general population (Diaz et al. 2013), and can be linked to broad cognitive traits, such as tendencies toward rumination (Andrews-Hanna et al. 2021), creativity (Raffaelli et al. 2023), and inattention (Kérébel et al. 2024), each measured by the relevant questionnaires.

In the present study, we focused on the durations of coherent thought segments. Earlier studies estimated thought lengths of 5–40 s (Klinger 1978, Pope 1978, Sripada and Taxali 2020, Li et al. 2021, Garg et al. 2025). We sought to characterize the distribution of thought segment durations (TSD), beyond their overall mean frequency. We reasoned that this could give us insights into the cognitive mechanisms underpinning thought transitions, as classical computational modeling has repeatedly shown that the timing of events provides information on the underlying processes (Posner 1986, Luce 1991, Schurger et al. 2012). To do so, we used a procedure of “online thought segmentation” (Pope 1978, Li et al. 2021, Garg et al. 2025) that enabled us to study the dynamics of spontaneous thought without interfering with its content.

We present a gradient of four tasks designed to progressively bring participants to segment their inner stream of thought. Starting from spoken aloud spontaneous word production (“Words” condition), they were introduced to the notion of thought segmentation while still producing words aloud (*Words + Online Segmentation*), before moving to silent word production (*Inner Words + Online Segmentation*) and ultimately to inner thought segmentation (*Inner Thoughts + Online Segmentation* condition). The last condition was our condition of main interest: participants let their minds wander and pressed a key whenever they noticed a change in the topic of their thought. The task gradient started with quantifiable semantic content, allowing us to ensure the validity of online reports of thought transitions. Indeed, it is believed that spontaneous thought is constrained and structured by semantic relations (Bar et al. 2007, Mildner and Tamir 2019), so thought transitions and semantic metrics should align when both are collected. The other end of the task gradient (segmented spontaneous thought) allowed us to measure the duration of thought segments with minimal interference. We show that the durations of coherent thought segments are best modeled with Wald distributions, suggesting an underlying accumulation-to-bound mechanism. In addition, we relate the parameters of this mechanism to questionnaire measures of participants’ tendency toward disorganized thought and non-clinical ADHD-like symptomatology. We find that the transition threshold is negatively correlated with these two questionnaire-assessed traits.

## Methods

### Participants

Sixty-one people from the general population participated in this study. The mean age was 25.3 years (SD = 6.7), and the sample was composed of 20 men and 41 women. They responded to a recruitment announcement sent through a dedicated mailing list. The inclusion criteria were the following: having normal or corrected to normal vision, being a native French speaker, and being 18 years old or older. This study received approval by the ethics committee of Université Paris Cité (CER-U-Paris Cité). All participants received a compensation of 15€ for 1 h 30 min of participation. The sample size was chosen so as to match the one of a previous experiment that used a comparable paradigm (Kérébel et al. 2024). Because no prior effect size estimates were available for this paradigm, no *a priori* power analysis was performed. The sample size was therefore determined pragmatically and chosen to be of a similar magnitude as the few published studies using comparable methods (Troyer et al. 1997, Sripada and Taxali 2020, Andrews-Hanna et al. 2021, Raffaelli et al. 2021, Martz et al. 2022, Kérébel et al. 2024). We also conducted an *a posteriori* power analysis. We used the “pwr” R package (Champely 2023) to compute the minimal observable Cohen’s d and Pearson’s r given a sample size of 60 participants, with a power of 0.80 and an alpha of 0.05. This resulted in a minimal d of 0.37 and a minimal r of 0.35. We approximated the corresponding log-odds using the following formula: log-odds = d ^*^ pi/sqrt(3) (see Chinn 2000). This resulted in minimal log-odds of 0.67.

The post-experiment debriefing session revealed that two participants had misunderstood the instructions in one or all conditions. The data of the corresponding trials were removed prior to the analyses. One participant who indicated only one thought transition in the whole experiment was excluded for the modeling analyses. In addition, some of the audio data went missing due to a technical problem (two participants for the control task, one for most of the main experiment).

### Materials and procedure

#### Tasks and design

##### Main tasks

**Figure 1:**
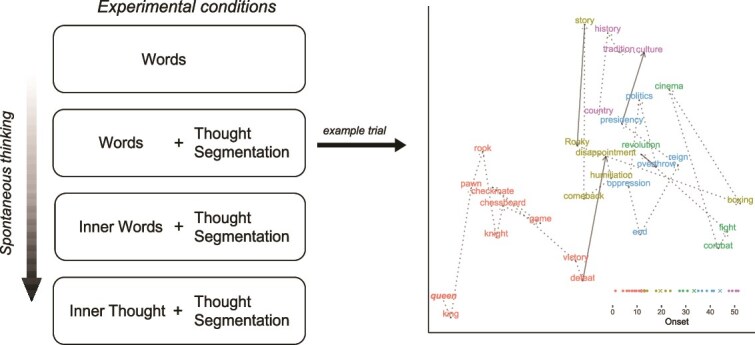
Illustration of the task. Left. Representation of the four conditions of the main task, forming a gradient toward probing spontaneous thinking. “Thought Segmentation”: Online Segmentation (OS). Right*.* Example series of words from *Words + OS* condition represented in a 2D semantic space (t-SNE reduction of the 300D word embedding model we used for the analyses). The first word produced is written in a bold and italic font. The insert plots represent the word onset in seconds. Colors: segmentation from the retrospection annotation procedure. Solid arrows: online segmentation (key presses).

Participants completed three blocks of four tasks forming a gradient towards probing pure thought segmentation: (i) *Words*, (ii) *Words + Online Segmentation*, (iii) *Inner Words + Online Segmentation*, (iv) *Inner Thought + Online Segmentation* (see Fig. 1).

In the “Words” condition they were instructed to say aloud the words that came spontaneously to their mind, at a rhythm that “felt natural to them”. In the *Words + Online Segmentation (OS)* condition they had to produce words exactly like in “Words” but also to press the spacebar whenever they realized they had just changed their thought topic (see example trial on Fig. 1). In the *Inner Words + OS* condition, the instructions were to do the same thing as in *Words + OS* without vocalizing the word, but saying them internally and performing the online thought segmentation with keypresses. Finally, in the *Inner Thoughts + OS* condition, participants just had to let their thoughts wander freely and to press the spacebar when they noticed a thought transition. This condition is the one that most directly measured what we were interested in, i.e. the segmentation of spontaneous thought in time. Importantly, since there was no constraint on the kind of words to produce nor on the pace of word production, the spontaneous word generation conditions are better thought of as indirect approximations of participants’ streams of thought rather than as a semantic fluency task. Our task can be seen as a continuation of previous work using word-based tasks for studying thought processes (Gray et al. 2019, Andrews-Hanna et al. 2021, Martz et al. 2022).

Previous observations in a former study (Kérébel et al. 2024) showed that the notion of changing thought topic during free word production reflects most people’s subjective impression in this context. We chose the wording of the instructions so that the online segmentation was not presented as a semantic exercise. In particular, we precised that “some words may seem closer to some people than to others, and there may be words that ‘go together’ for [them] for personal reasons” (see the instructions script in the Supplementary materials, Task instructions section).

In the first block, participants read the instructions of each condition before doing the corresponding task. The experimenter was present during training and made sure that the instructions were well understood. In the two following blocks, they completed the four tasks in a row. At the beginning of each trial, a neutral seed word was displayed on the screen, and participants had to read it aloud. The list of seed words is reported in the Supplementary Materials. It was made clear that the goal of the task was not to keep revolving around these seed words. After 3 s, the seed word was replaced by a fixation cross that remained until the end of the task. Each trial lasted 120, 150, or 180 s, with the order shuffled within conditions. At the end of each trial, participants answered two questions about the intensity of their thought on a continuous scale (see Supplementary Materials).

##### Control tasks and subjective annotation

After the three main blocks, participants did two trials of an “Online Segmentation Control task”. In this task, we presented words one by one on the screen, and they had to press the spacebar to indicate the groups of words that seemed to belong together. The words were taken from real trials of a pilot participant and were presented at the same pace at which they had originally been produced. At the end of the experiment, participants were presented with a printed transcription of the words they had produced in the task (conditions “Words” and *Words + OS*). They were instructed to indicate the groups of consecutive words that corresponded to a single, coherent thought (Kérébel et al. 2024). This subjective annotation procedure provided us with a retrospective “offline segmentation” of the series of words. We introduced this retrospective offline segmentation to better understand the online segmentation that reflects thought segmentation more directly than the retrospective one. Participants also did a similar exercise on two series of words that they had not produced, as a control (“Offline Segmentation Control”). The series of words used in both control tasks were the same for all participants and are reported in the Supplementary Materials.

#### Set up

Participants were seated in front of a computer screen (52.3 cm^*^ 29.5 cm), at a 70 cm distance. Their head was stabilized by a forehead-rest and a chin rest. Text and stimuli were displayed in white on a gray background. They were instructed to avoid moving or blinking and to keep fixing the center of the screen as much as possible during the task.

Pupillometric data was recorded with an EyeLink 2000 eye-tracker (SR Research). These data were not used in the current study. Audio data was captured using the Audacity software, and transcribed and timestamped using the Vosk Speech Recognition Toolkit (Shmyrev and Alpha Cephei 2020) in Python. We used the “fastText” embedding model (Grave et al. 2018) to compute semantic distances between the words.

#### Questionnaires

Participants filled two questionnaires between the main task and the subjective annotation procedure. First, they completed the ADHD Self-Report Scale (ASRS, Kessler et al. 2005, French translation by Caci et al. 2014), a clinical questionnaire used by psychiatrists to help diagnose ADHD (see Supplementary Materials). Since we tested participants from the general population, we take the score of this questionnaire to reflect their “attentional profile”, i.e. trait tendency for inattention and impulsivity. We used the short version of this questionnaire (ASRS-A) that has been shown to be a better diagnostic tool than the full one (Kessler et al. 2005). Second, they completed the Mind Excessively Wandering Scale (MEWS, Mowlem et al. 2019, the French translation we used is reported in the Supplementary Materials). This recent questionnaire more specifically assesses the construct that our task was targeting, namely the tendency for disorganized thought.

### Analyses

#### Words data preprocessing

The onset time of each word was obtained automatically via Vosk (Shmyrev and Alpha Cephei 2020), then manually checked and corrected. Spelling, plural forms and capital letters were manually corrected. The preprocessed data file is available on the Open Science framework (OSF, https://osf.io/qc5fj/).

#### Statistical analyses

All statistical analyses were performed using *R* (version 4.3.3). For assessing the relationship between two variables, we used correlations when there was one point per participant, and mixed effect—or generalized mixed effect—models otherwise. The mixed effect-models were fitted using the *lme4 R* package (Bates et al. 2015). In terms of random intercepts, we included the *subject id.* for the models fitted on data averaged by condition, and the trial id. nested in subject id. (series *subject id./series nb.*) for the models fitted on the full data. In order to avoid false positives, we included the maximal number of random slopes justified by the design and supported by the data (Matuschek et al. 2017). We used likelihood ratio tests to systematically check that our models of interest outperformed null models including only the slopes.

We report p-values as estimated using Satterthwaite’s degrees of freedom method with the *lmerTest R* package (Kuznetsova et al. 2017). In the Supplementary Materials we report the tables of every models, with 95% confidence intervals and effect sizes in the form of R-squared for linear models (*MuMIn R* package, implementing the methods proposed by Nakagawa and Schielzeth 2013) and odd ratios for logistic models. In order to assess evidence for null effects, we computed Bayes factors or approximation thereof. For correlations, we used the *BayesFactor R* package (Morey et al. 2012) with default priors to compare a regression with and without the predictor of interest. For mixed-effect models we approximated it from the Bayesian Information Criterion (BIC): BF_10_ = exp((BIC_0_ − BIC_1_)/2) (Wagenmakers 2007). The scripts used to produce the analyses, tables and figures reported here are available on OSF, along with the Supplementary Materials and the data.

#### Metrics

Our main measure of interest was the time interval between consecutive keypresses, or TSD. We also characterized word production using both semantic and temporal measures. For the semantic measure we used a pretrained word embedding model of French (“fastText”, Grave et al. 2018) that associates each word with a vector in a 300-dimensional space. This model had been successfully used on similar data (Kérébel et al. 2024). We computed a semantic distance (thereafter “consDist”) between consecutive words based on the cosine similarity of the vectors of those words in the embedding model: consDist(A,B) = acos(cosine(A,B))/pi. This metric is called the angular distance. For some of our analyses, it made more sense conceptually to work with semantic distances, as opposed to semantic similarities. Contrary to the often-used cosine distance (cosine distance = 1—cosine similarity), the angular distance is a proper distance metric in terms of geometric properties. Note that apart from very similar words, angular distance ~ −1^*^cosine similarity. Our temporal measure was the time interval between consecutive word onsets, or word onset asynchrony (thereafter WOA).

## Results

We proceeded in two steps for analyzing the data. In the first part, we validated the online segmentation as a measure of thought transitions. In order to do that, we compared behavior between conditions, and we tested the coherence of self-reported thought segmentations with questionnaire scores and objective measures. In the second part, we set out to identify a potential cognitive mechanism underlying thought transitions, moving from a descriptive to a process account of individual differences.

### Online segmentation reflects thought transitions

#### Similarities of behavior between conditions

Summary statistics of behavior in the four conditions are presented in Table 1 and Supp. Tables 1 and 2. We first compared behavior across conditions by means of paired t-tests. We considered the time intervals between consecutive words (WOA) and the time durations between consecutive keypresses, i.e. the TSD. The pace of word production (mean WOA) did not differ statistically between the *Words* and *Words + OS* conditions (t(58) = −0.69, 95% CI [−0.21; 0.1], *P* = .49, d = 0.09). Participants indicated longer TSD in the *InnerThought + OS* condition compared to *Words + OS* and *Inner Words + OS* (t(54) = 2.59, 95% CI [0.68; 5.31], *P* = .037, d = 0.35, and t(54) = 4.53, 95% CI [2.46; 6.37], *P* < .001, d = 0.61 respectively). There was no statistical difference between the mean TSD from the Words+OS and Inner Words+OS conditions (t(56) = 2.15, 95% CI [0.1; 2.78], *P* = .11, d = −0.28). Offline, participant identified more segments in the “Words” than in the *Words + OS* condition (t(58) = 3.5, 95% CI [0.17; 0.64], *P* < .001, d = 0.46).

**Table 1:** Summary statistics of word production and thought segmentation. Format: mean (sd). WOA: Word Onset Asynchrony, TSD: Thought Segment Durations.

	WOA (sec.)	TSD (sec.)	nb. online segments/min.	nb. offline segments/min.
*Words*	2.64 (1.16)			4.06 (1.73)
*Words + OS*	2.7 (0.92)	17.47 (7.82)	4.11 (2.15)	3.66 (1.41)
*Inner + WordsOS*		15.91 (7.56)	4.62 (3.04)	
*Inner + ThoughtsOS*		20.33 (10.16)	3.83 (3.01)	

We then tested whether the segmentation metrics correlated between conditions and type (online/retrospective) by looking at the correlations between them. All correlations were significant and positive (rho > 0.3, *P* ≤ .01), except between the “Words” condition and the *InnerThought + OS* condition (*P* = .05). The full correlation table can be found in the Supplementary Materials (Supp. Table 3).

Finally, we tested whether the online segmentation aligned with the self-reported tendency toward disorganized thought. In the *InnerThought + OS* condition we observed a negative correlation between the mean TSD and the MEWS score (rho = −0.33, *P* = .01), but no significant correlation between mean TSD and ASRS score. There was no significant correlation in the Words+OS and InnerWords+OS condition (all *Ps* > .05). The full correlation table can be found in the Supplementary Materials (Supp. Tables 4 and 5).

#### Keypresses reflect thought transitions

We assessed the validity of the Online Segmentation (keypresses) as a measure of thought segmentation, using the *Words + OS* condition in which we had both the thought segmentation and a proxy for the stream of thought.

We started by quantifying the correspondence between online and offline segmentation. First, we used a linear mixed effect model to predict the number of keypresses in each trial based on the number of retrospectively identified segments, with the number of words as a control. There was a positive effect of the number of segments (est. = 0.82, *P* < .001), and no significant effect of the number of words (*P* = .52, see Supp. Table 6). Second, we used permutations to verify that the online and offline segment boundaries aligned (mean distance between online and offline boundaries = 1.66 +/− 0.71 words, mean chance level at 2.65 +/− 1.08, see Supp. Analysis 1).

We then investigated whether the keypresses reflected meaningful transition points in mental content. We predicted whether each word was immediately following a keypress or not in a hierarchical logistic regression, with the consecutive semantic distance (thereafter “consDist”), the WOA and word onset time in the trial as predictors (see Supp. Table 8). We observed a positive effect of the WOA (est. = 0.37, *P* < .001) but no significant effect of the consDist (*P* = .41), nor of word onset time (*P* = .29).

These elements suggest that participants reported meaningful transitions, as the online segmentation corresponded to temporal boundaries and mirrored retrospective judgments.

#### Thought transitions are driven both by semantics and elapsed time

Then, we aimed at better understanding why and when participants reported thought transitions, again using the *Words + OS* condition.

We then sought to better understand when and why participants reported thought transitions, again on the *Words + OS* condition. To this end, we fitted logistic regression models predicting whether a given word immediately “preceded” a keypress (i.e. a reported transition) or not. We emphasize that this analysis is conceptually distinct from the previous one. While the earlier model (section 3.1.2) characterized properties of reported thought transitions (i.e. semantic jumps and duration) by analyzing words following a keypress, the current analysis focuses on predicting the occurrence of these transitions based on the words preceding them.

We wanted to assess the extent to which thought transitions were based on thought content, so we started by focusing on semantic variables. We included three theory-informed semantic predictors: the semantic distance with the previous word (consDist), the accumulated consDist since the previous keypress (cumConsDist), and the semantic distance with the word following the last keypress (lastKPDist). These variables were selected to disentangle the relative contributions of local changes (i.e. word-to-word dynamics) and more global changes occurring over longer sequences on participants’ subjective reports of transitions. More specifically, the lastKPDist can be understood as a bird’s flight distance in semantic space since the last transition, while the cumConsDist would be the path distance and consDist represents a local change. We observed positive main effects for the three predictors (respectively est. = 2.69, *P* < .001, CI = [1.62; 3.76]; est. = 0.11, *P* < .001, CI = [0.07; 0.15]; est. = 3.07, *P* < .001, CI = [1.77; 4.36]).

In order to test potential effects of temporal variables, we compared the previous model with models where each semantic predictor was replaced by its temporal counterpart: WOA for the consDist, time elapsed since the last keypress (nSec) for the lastKPDist and the cumConsDist. Note that we replaced semantic variables by temporal variables instead of simply adding the temporal variables to the first model in order to avoid collinearity issues.

Further, we compared the candidate model with a simplified version without one of the predictors. We compared the models based on the BIC and the Akaike Information Criterion, two standard metrics that take into account the number of model parameters, in order to avoid overfitting.

We found that a model including the consecutive semantic distance with the previous word (consDist), the semantic distance with the word following the last keypress (lastKPDist), and the time elapsed since the last keypress (nSec) was the best fit to our data (respectively est. = 2.66, *P* < .001, CI = [1.59; 3.73]; est. = 2.86, *P* < .001, CI = [1.38; 4.35]; est. = 0.02, *P* < .001, CI = [0.02; 0.03], see Supp. Table 10). An exhaustive list of the models we compared can be found in the Supplementary Materials (Supp. Table 12).

### Thought segment durations follow a Wald distribution

#### Model space

**Figure 2:**
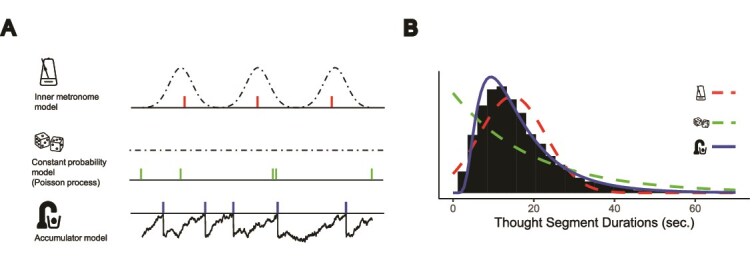
Illustration of the model comparison. (A) Schematic representation of the model space. For the Accumulator model we only represented the version with two parameters for conciseness. (B) Distribution of TSD, normalized to the mean for each participant, and density probability functions of the candidate models. Note that the fitted probability functions presented here are mainly illustrative, since the analyses we report were done on a participant basis.

Next, we set out to identify the cognitive mechanism underlying thought segmentation, even in our target condition (*InnerThought + OS*) where, by design, no semantic data was available. We used the distribution of TSD (see Fig. 2) to constrain the possible underlying mechanisms. We identified three plausible candidate mechanisms and corresponding models. In the “Constant probability” model, the probability to press the key is the same at every time point (a Poisson process). In the “Inner metronome” model, the duration between key presses is roughly constant as if an inner metronome regulated the timings of thought transitions, with Gaussian noise. In the “Accumulator” models, keypresses are driven by an accumulation-to-threshold mechanism (Wald model) or an accumulation-to-threshold with a non-accumulation time (shifted-Wald model). The models are recapitulated in Supp. Table 20, and illustrated on Fig. 2.

#### Model comparison

The four candidate models were fitted on the data of each participant individually (*Words + OS, InnerWords + OS*, and *InnerThought + OS* conditions pooled), using minimum likelihood estimation (Delignette-Muller and Dutang 2015, R Core Team 2024). We compared model fits on the basis of the BIC (Schwarz 1978). Results are summarized on Table 2. The accumulation model without a non-decision time parameter was the best for most of the participants. We did the same comparison and model estimation on the online segmentation control task. The Wald distribution was the best fitting 1 for 54 participants (see Supp. Table 21).

**Table 2:** Results of the model comparison. Models fitted on TSD. *Words + OS, InnerWords + OS*, and *InnerThought + OS* conditions pooled. For each line, “nb. best” indicated the number of participants whose data was best fitted by the corresponding model

Model	nb. best
*Accumulator*	46
*Inner metronome*	10
*Accumulator + non-accumulation time*	2
*Constant probability*	2

**Figure 3:**
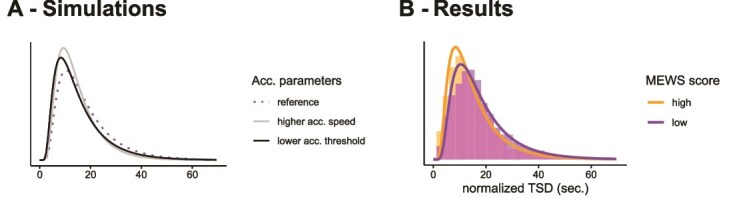
Visualization of the pattern of relationship between the parameters of the accumulation model and the questionnaire scores. (A) Visualization of the change in the distribution of TSDs caused by an increase of the accumulation speed (grey) or a lowering of the accumulation threshold (black), compared to a reference level (purple). (B) Distributions of TSDs depending on the MEWS score (median split). The two distributions are shown for illustrative purposes; the analyses were conducted using the continuous questionnaire scores. *Note*: The purple distribution is the same on the A and B panels.

The winning accumulation model has two parameters. The accumulation rate (gamma) reflects the speed at which an internal signal builds up over time, whereas the threshold (alpha) corresponds to the level this signal must reach to trigger a thought transition. Figure 3 illustrates how model parameter values influence the resulting TSD distribution.

Importantly for the analyses that follow, the 12 participants whose data were not better fitted by the Wald or shifted-Wald distributions did not display any particular tendency in the questionnaires. We tested this by comparing the distributions of questionnaire scores between these 12 participants and the others (Kolmogorov–Smirnov and Wilcoxon Rank-Sum tests, all *Ps* > .3).

Following good practices in computational modeling (Palminteri et al. 2017), we performed parameter recovery and model recovery tests. Parameters fitted on a simulation strongly correlated with the true parameters (alpha: r = 0.83, *P* < .001; gamma: r = 0.73, *P* < .001 for the winning accumulator model), and model recovery was also very high (accumulator: 99%; constant probability: 99%; inner metronome: 85%). Details on our fitting procedure, quality checks and additional analyses can be found in the Supplementary Materials (sections Parameter recovery, Model recovery, and Outlier fits).

### Accumulation thresholds correlate with inattention and tendency toward disorganized thought

We then turned to better characterizing individual differences in thought segmentation by investigating whether the questionnaire scores were selectively predictive of one of the estimated parameters of the “accumulator” model on all participants.

First, we looked at the parameters estimated for each participant on the three conditions providing online segmentation (Words+OS, InnerWords+OS, and InnerThought+OS). We observed negative correlations between the threshold and the questionnaire scores (ASRS: r = −0.41, *P* = .002, BF = 28.31; MEWS: r = −0.31, *P* = .018, BF = 3.8), but not with the accumulation rate (ASRS: r = −0.2, *P* = .12, BF = 0.87; MEWS: r = 0.008, *P* = .95, BF = 0.3).

Then, we focused on our condition of main interest. We fitted the accumulation model on the *InnerThought + OS* condition only and observed a similar pattern: there were significant negative correlations between questionnaire scores and model thresholds (ASRS: r = −0.32, *P* = .019, BF = 3.8; MEWS: r = −0.35, *P* = .011, BF = 5.9), but not between questionnaire scores and model accumulation rates (ASRS: r = −0.055, *P* = .7, BF = 0.33; MEWS: r = 0.133, *P* = .34, BF = 0.47). For completeness, correlations for each condition fitted individually are reported in the Supplementary Materials (Supp. Table 23).

Crucially, the parameters fitted on the online segmentation control task did not correlate with the questionnaire scores (all *Ps* > .25, all BF*s* between 0.3 and 0.6).

## Discussion

We studied the duration of coherent thought episodes by asking participants to report their thought transitions in real time. First, we found a positive correlation between participants’ self-reported tendency toward disorganized thought and the frequency thought transitions observed in the laboratory. Next, we showed that the distribution of TSDs revealed the signature of an accumulation-to-threshold process. We further found that the shorter coherent thought segments in individuals with a greater tendency toward disorganized thought—and associated with a higher trait inattention—is explained by a lower threshold in this accumulation process.

### Interpretation of the results

Prior to interpreting our results, it is necessary to address the degree of spontaneity of the thoughts captured during the experiment. We argue that the *InnerThought + OS* condition is at least as unconstrained as the think-aloud paradigm frequently employed in spontaneous thought research (e.g. Raffaelli et al. 2021), which requires both a continuous monitoring and a verbalization effort. Our approach may be viewed as a minimal version of self-caught experience-sampling—a methodology widely utilized in mind-wandering studies. Within this framework, it is generally accepted that the integration of self-caught probes does not fundamentally impede the flow of spontaneous thought (see Chu et al. 2023 for a review).

We observed a mean duration of coherent thought segments of 20.3 s. This can be directly compared to the results of a handful of previous investigations where TSDs were quantified. The studies which used a think-aloud paradigm with an external segmentation from raters all found estimations of ~20–30 s for the duration of thought segments (Pope 1978, Sripada and Taxali 2020, Li et al. 2021, 2023, Raffaelli et al. 2021, Garg et al. 2025). Three studies complemented this method with a task where participants let their mind wander freely and indicated thought changes in real time, yielding thought durations of 5 s (Pope 1978), 30 s (Sripada and Taxali 2020, Garg et al. 2025), and 33–40 s (Li et al. 2021). Note that the instructions in Pope’s experiments required to report any change of conscious experience, not thought transitions *per se*, which probably explains the shorter durations they observed. Interestingly, Klinger (1978) found similar durations of ~9–15 s when probing individuals to report the length of their last thought segment. All these durations are comparable to indirect estimates of the duration of “mental states”: e.g. Tseng and Poppenk (2020) derived durations of 9 s from neural data in resting state fMRI, Bastian and Sackur (2013) inferred durations of 10–20 s from behavioral data.

Beyond the overall frequency of transitions, our protocol provided us with the full distribution of TSDs, enabling us to try to identify the most likely mechanism from a set of plausible options. Our results suggest that, in a situation where external distractions and proximal goals are reduced to a minimum, the timing of thought transitions is driven by an accumulation-to-threshold mechanism. The data further reveal that participants higher in trait inattention or with a higher tendency toward disorganized thought have more frequent thought transitions because of a lower threshold in this process.

Our proposed mechanism for thought transitions complements and potentially extends existing models. These models, often derived from research on semantic fluency tasks, typically describe thought trajectories as resulting from either random walks or foraging processes within a semantic space (see Mildner and Tamir 2019 for a review). Our study addresses a distinct question: we focus on the subjective experience of transitions in the stream of thought, rather than on a continuous movement within a semantic space. From a modeling perspective, it is plausible that introducing a threshold mechanism into semantic search models could produce distributional properties similar to those we observed. In this sense, our proposed mechanism is complementary to approaches inspired by the semantic fluency literature, while extending beyond purely semantic contexts.

Now, the questions of “what” would be accumulated, and at which cognitive level, remain open. We approached it by considering that the spontaneous word production conditions provide an approximation of a participants’ thought trajectory in a semantic space. We found a semantic drift preceding thought transitions: as participants got closer to a transition, the bird flight distance to the start of the episode progressively increased. However, the time elapsed since the last transition proved to be a better predictor of the next transition than either the accumulated semantic distance along the trajectory or the number of words produced. This suggests that the accumulated variable also integrates another component, potentially non-semantic in nature. This interpretation would be supported by the similarities between our online segmentation procedure and Libet’s task (Libet et al. 1983), where participants are required to press a key whenever they “feel the urge to move”. It has been suggested that, in this paradigm, movement initiation results from an accumulation of neural noise that can be observed in the EEG signal (Schurger et al. 2012), resulting in a distribution of waiting durations with a shape similar to the one we observed. As participants in Libet-like tasks may feel the mounting pressure of an urge to move with the increase of accumulated neural noise until the reaching of a threshold, participants in our task may transition to a new thought episode as a result of a mounting pressure of an “urge to explore new thoughts”. Note that our *InnerThought + OS* condition still differs from Libet experiments in several important aspects. In particular, the mental events we asked participants to monitor exist outside of the context of the experiment, and are arguably easier to introspect and more familiar to them than an “urge” to press a key.

Our results suggest revisiting the classical notion of the train of thoughts as governed by associations of ideas (Hobbes 1651/2008) and external distractions (Song and Wang 2012, Plimpton et al. 2015). Indeed, these mechanisms alone should lead to a Poisson-like process if the probability of a concept being a pivot for a new chain of thoughts and the probability of an external distraction were constant. Our results suggest that underneath these mechanisms, there exists an accumulation process leading to thought transitions even without external or semantic distraction. Further work is needed to establish whether and how the two levels interact. For example, it could be that the distance to the accumulation threshold influences distractibility, i.e. the likelihood that a distractor would trigger a thought transition.

The link between ADHD traits and higher exploratory behaviors has recently been interpreted as a consequence of increased internal noise (Van den Driessche et al. 2019, Addicott et al. 2020, Dubois and Hauser 2020). Our finding that trait inattention and the tendency toward disorganized thought are associated with lower transition thresholds is congruent with other results from the literature and suggests a complementary, explanatory pathway for ADHD traits. Given that ADHD is associated with trait boredom (Hsu et al. 2020) and novelty seeking (Donfrancesco et al. 2015), we propose that the more frequent thought transitions observed in our study could result from lower thresholds in accumulation processes inducing a lower tolerance for boredom. It is worth noting that a low accumulation threshold is typically interpreted as a marker of impulsivity. Our results align with a previous study, where we found that both thought segmentation and tendency for impulsive exploration correlated with trait inattention (Kérébel et al. 2024). Considering that recent accounts frame mind-blanking as a transitory state (Boulakis and Demertzi 2025), our results are also consistent with previous work showing that ADHD is associated with a higher rate of mind-blanking reports (Van den Driessche et al. 2017).

### Alternative interpretations

In principle, the distribution of TSD could exhibit the signature of an accumulation process in two different scenarios. The one we implicitly favored so far would be that there exist “true” thought transitions that participants noticed and reported, and that these are driven by a latent accumulation mechanism. Another one would be that mental content was drifting without clear boundaries, and that participants relied on an accumulation of small differences to segment it, as a result of task demands. Four elements suggest that the first scenario is more likely. First, several empirical studies found results supporting the view of a segmented—as opposed to continuous—stream of thoughts (Sripada and Taxali 2020, Raffaelli et al. 2023, Kérébel et al. 2024). We can also make a parallel with research on memory, where recent results challenge the view of a continuous and passive drift of mental context (DuBrow et al. 2017). Second, in the condition with words pronounced aloud, we observed a transient increase in consecutive semantic distance prior to the keypresses, suggesting that the reported thought transitions were salient (see Supp. analysis 2 and Supp. Fig. 2). Third, the online segmentation control task singles out the decision component (since participants are not producing the series of words they segment) and the fitted accumulation threshold did not correlate with the questionnaires. This suggests that the relationships we observed in the main task reflect intrinsic properties of the thought transitions (*de re*), not of the detection (*de dicto*). Fourth, if the second scenario were true, online and offline thought segmentations would be independent, especially between conditions where only one of the two was performed. Yet we found positive correlations between offline segmentation in the “Words” condition and online segmentation in the *Words + OS* and *InnerWords + OS* conditions, and Li et al. (2021) reported a similar observation.

There remains the possibility that the inter-individual segmentation differences we observed correspond to differences in introspection capacity: some people would experience more variable trains of thought not because their thought changes more, but because they are better at noticing when it changes. In a sense, this is as much a limitation of our design as a limitation of our current understanding of thought in general. However, if this was the case, we could expect participants with higher trait inattention to exhibit less detections. Yet, one could then further argue that these people appear inattentive to their environment precisely because they pay more attention to their thought process. In sum, this is a valid point that would require further investigation.

### Limitations

We acknowledge several limitations in the present study. First, since we asked participants to report only punctual thought transitions, our experiment is by design blind to the potential presence of progressive transitions, mind-blanking episodes, or unfocused thought moments, that we had observed in a previous study (Kérébel et al. 2024). Second, we did not consider all possible sources of inter-individual variability. In particular, post-experiment debriefing revealed that for some participants word production stimulated thought processes, while for others it was inhibiting. Our conditions formed a gradient progressively guiding participants toward free thinking, but the cognitive demands of each condition did not necessarily follow the same gradient. Another source of inter-individual variability would be the question of what different participants consider to constitute a transition between thoughts. Within the conceptual framework that we based our research on, this would amount to the threshold on the step size for a walk on a semantic network. Investigating this issue is an exciting avenue of research. Third, all the models we considered suppose that the duration of each thought segment is independent of the previous one, yet this is not the case since TSDs are partially autocorrelated (see Supp. Analysis 6). Fourth, because all participants completed the four conditions in the same fixed, progressive order, our design may have introduced carryover effects. In particular, participants may have developed a bias toward verbal thinking in the inner thinking condition, or adopted a more segmented mode of thought as a consequence of the preceding tasks. Although it is inherently difficult to assess the precise nature of participants’ experiences during the inner thinking condition, we attempted to gain some insight through semi-structured debriefings conducted at the end of each session. Most participants reported that this condition felt easier than the others, primarily because they were not required to generate words. Out of the 18 participants for whom the full debriefing was available (due to a lack of time for some participants), only one reported that they innerly produced series of words during the InnerThought+OS condition. This suggests that they were not simply continuing the silent word generation task during this phase, although we cannot fully rule out more subtle carryover influences. Fifth, while we did not observe any correlation between the accumulator model’s speed parameter and questionnaire score, this absence of observed effect cannot be taken as evidence for an absence of relationship. For example, it could be that the two constructs covary for some participants but not for others based on a third factor, or that the relationship exists but is too small to be observed in our study. Future work could address this question by combining the inner thinking segmentation paradigm with a set of battery tests and complementary questionnaires.

## Supplementary Material

Supplementary-material_niag045

## Data Availability

Preprocessed data and analysis scripts are available at https://osf.io/qc5fj/.
