# Supplementary material for: Dynamics of the stream of thought: insights from an accumulation-to-threshold model
Source: Neurosci Conscious. 2026 Aug 14;2026(1):niag045. doi: 10.1093/nc/niag045 (PMC13474264; doi:10.1093/nc/niag045)
Supplement: Supplementary file 1 [file supplementary-material_niag045.zip › rev._1_-_Revised_Supplementary_materials_-_KEREBEL_SACKUR_niag045.docx]

Dynamics of the Stream of Thought: Insights from an Accumulation-to-Threshold Model

Supplementary materials

*Adrien Kérébel^1, †^, Jérôme Sackur^1,2^*

*1. Laboratoire de Sciences Cognitives et Psycholinguistique (LSCP), Département d’Études Cognitives de l’École Normale Supérieure (ENS), Centre National de la Recherche Scientifique (CNRS), École des Hautes Études en Sciences Sociales, Paris Sciences et Lettres (PSL) Research University, Paris, France.*

*2. Ecole des Hautes Etudes en Sciences Sociales (EHESS)*

*†. Corresponding author:* [adrien.kerebel@gmail.com](mailto:adrien.kerebel@gmail.com); 29 rue d’Ulm, 75005 Paris, France

# 1. Task materials and questionnaires

*Task instructions*

During the first block, participants read the following instructions on the screen (translated from French). The experimenter made sure the instructions were well understood, rephrasing if needed.

- *Words* condition: “For 2 to 3 minutes, you'll have to say all the words that come spontaneously to your mind, one after the other. To get you started, a word will appear on the screen just before you start. When a cross appears, say that word, then any others that come to mind, until further instructions are displayed. During the series, keep your eyes on the center of the screen as much as possible. The word that appears on the screen is not a theme, but the starting point for your series of words.”
- *Words + OS* condition: “You may have noticed that when you produce a series of words, some words ‘go together’, i.e. they correspond to a same thought. In the ‘Word and keypress’ series, you will say the words that come spontaneously to mind, and press the SPACE key each time you realize that you have changed the group of words that ‘go together’. To get you started, a word will appear on the screen just before you start. When a cross appears, you say that word, then any others that come to mind, and press the SPACE key each time you change word groups. There's no right answer! Some words may seem closer to some people than to others, and there may be words that ‘go together’ for you for personal reasons.”
- *Inner Words + OS* condition: “This is almost the same task as the in the ‘Word and keypress’ series, but this time you will say the words in your head, without saying them out loud. In the ‘Inner word and keypress’ series, you'll say the words that come spontaneously to mind in your head, and press the SPACE key each time you realize you've changed the groups of words that ‘go together’. To get you started, a word will appear on the screen just before you start. When a cross appears, you'll say that word out loud, then mentally say all the others that come to you, and press the SPACE key each time you change word groups. There's no right answer! Some words may seem closer to some people than to others, and there may be words that ‘go together’ for you for personal reasons.”
- *Inner Thought + OS* condition: “In the ‘Thought and keypress’ series, you won't produce a series of words. You'll let your thought wander freely, and press the SPACE key whenever you realize you've changed thought topic. To get you started, a word will appear on the screen just before you start.”

*Seed words*

The seed words were drawn from a list of words filtered for frequency, context availability and arousal (Bonin et al., 2013; New et al., 2004). We chose words that every participant should know, that were tangible enough to trigger a train of thought, and that were neutral in arousal level. Each participant saw the same set of seed words per condition, in a randomized order.

Seed words used by condition (translated from French):

- Words: *brother, hour, cake*

- Words+OS: *plane, city, hand*

- InnerWords+OS: *joy, hat, queen*

- InnerThoughts+OS: *rain, farm, suitcase*

*Thought probes* (translated from French)

Q1: “To what extent were your thoughts present or intense right before this screen appeared?” (continuous slider: No thought – Very present/intense thoughts)

Q2: “To what extent did the presence or the intensity of your thoughts varied during this last trial?” (continuous slider: No variability – A lot of variability).

We were hoping to get both a local and global estimation of participants’ thought intensity, but it appeared that the understanding of the two questions and their relationship was not consistent across participants so we did not use them for analysis.

*Online Segmentation Control Task words*

Word were presented one by one on the computer screen.

#1: pâtisserie, chocolat, manger, plaisir, cuisine, table, convive, déjeuner, dessert, fromage, aliment, magasin, rue, épicerie, commerce, ville, déplacement, transport, bus, voiture, argent, monnaie, billet, pièce, téléphone, smartphone, réseau, communication, internet, application, parole, échanges, gens, citoyen, ami, copain, mot, verbe, culture, musée, tableau, objet, histoire, archéologie, instruction, culture, enrichissement, développement, sport, plein air, avion, effort, voyage, pays, paysage, montagne, randonnée, vu, neige, rencontre, échange, apprendre, regarde, regarder, accueil, image, perspective, couleur, élément, soleil, nature, lune, étoile, ciel, regarder, ordinateur, écran, sablier, temps, fin, blanc, noir, marque

#2: voyage, nuages, long, court, distance, pays, monument, état, président, américain, rêve, canada, italie, france, espagne, portugal, australie, Inde, russie, brésil, plage, carnaval, rio, statue, liberté, new york, washington, floride, miami, texas, western, film, acteur, eastwood, oscars, césars, festival, cannes, tapis, rouge, robe, costard, limousine, voiture, hôtel, plage, sable, mer, bateau, équipage, pirate, procès, fan, chanteuse, concert, musique, billets, vente, stade, public, étage, siège, haut, nourriture, carte, invitation, loge, miroirs, chaises, tenue, maquillage, cheveux, laque, armoire, canapé, sol, lumière, salle, mur, lampadaire, rue, soir, poubelle, photo, animaux, feu

*Offline Segmentation Control Task words*

Word series were presented in two vertical columns, on paper.

#1: gâteau, glaçage, pâtisserie, cerise, chocolat, sur, dessous, série, film, divertissement, suspense, temps, attente, micro, voix, chant, talent, don, Dieu, religion, juif, chrétien, musulman, occidental, asiatique, américain, nationalité, international, gouvernement, premier ministre, ministre, démission, mécontentement, population, démocratie, parlement, monarchie, système, capitaliste, anarchiste, mondialiste, écologiste, étudiant, manifestant, militant, politique, apolitique, peur, collapsologie, sociologie, ouvrier, salarié, patron, entonnoir, étuve, toilette, bidet, chasse d’eau, eau, bouteille, thé, froid, chaud, pas, mal, touche, toucher, doigt, tactile, digital, téléphones, tablette, ordinateur, hésitation, stylet, stylo, feutre, critérium, écrire, calligraphie, fin, film, cinéma

#2: voyage, transport, pays, déplacement, pollution, carbone, empreinte, envie, découverte, enrichissement, apprendre, culture, langue, différente, alimentation, cuisine, nourriture, eau, mer, paysage, animaux, poisson, repas, pâtes, viande, fromage, mousse, chocolat, gâteau, envie, sucre, table, convive, invité, discussion, parole, écoute, regarder, assiette, couvert, boisson, alcool, cocktail, verre, fenêtre, tour, immeuble, architecture, maison, jardin, animaux, ferme, paille, champ, tracteur, lapin, oiseau, nature, ruralité, rural, campagne, province, espace, naturel, vide, route, camion, marchandise, bateau, conteneur, remorque, achat, magasin, monde, système, développement, question, problématique, avenir, futur, triangle, carré, rond, violet, bleu, noir

*ASRS questionnaire* (French translation from Caci et al. (2014))

Instructions: *Veuillez répondre aux questions ci-dessous en vous évaluant vous-même sur chacun des critères indiqués à l’aide des mots situés sous chaque question. Répondez à chaque question en cliquant sur la case qui décrit le mieux ce que vous avez ressenti et comment vous vous êtes comporté au cours des 6 derniers mois.*

Options: *Jamais, Rarement, Parfois, Souvent, Très souvent*

Questions:

*1. À quelle fréquence avez-vous des difficultés à finaliser les derniers détails d’un projet une fois que le plus intéressant a été fait*

*2. À quelle fréquence avez-vous des difficultés à mettre les choses en ordre lorsque vous devez faire un travail qui demande une certaine organisation*

*3. À quelle fréquence avez-vous des difficultés pour vous souvenir de vos rendez-vous ou de vos engagements*

*4. À quelle fréquence avez-vous tendance à éviter ou à remettre à plus tard un travail qui demande beaucoup de réflexion*

*5. À quelle fréquence avez-vous la bougeotte ou agitez-vous vos mains ou vos pieds lorsque vous devez rester assis pendant un long moment*

*6. À quelle fréquence vous sentez-vous trop actif ou obligé de faire des choses, comme si vous étiez actionné par un moteur ?*

Original questions (English):

*1. How often do you have trouble wrapping up the final details of a project, once the challenging parts have been done?*

*2. How often do you have difficulty getting things in order when you have to do a task that requires organization?*

*3. How often do you have problems remembering appointments or obligations?*

*4. When you have a task that requires a lot of thought, how often do you avoid or delay getting started?*

*5. How often do you fidget or squirm with your hands or feet when you have to sit down for a long time?*

*6. How often do you feel overly active and compelled to do things, like you were driven by a motor?*

*MEWS questionnaire* (translated for this study from Mowlem, et al., 2019)

Instructions: *Vous trouverez ci-dessous un certain nombre d'énoncés concernant vos pensées. Pour chaque énoncé, choisissez la réponse qui décrit le mieux vos pensées en général. Il n'y a pas de bonnes ou de mauvaises réponses. Répondez en fonction de votre ressenti et de votre expérience personnelle.*

Options: *Pas du tout ou rarement, Parfois, La plupart du temps, Presque tout le temps ou constamment*

Questions:

*1. J'ai de la difficulté à contrôler mes pensées.*

*2. J'ai du mal à 'éteindre' mes pensées.*

*3. J'ai deux pensées ou plus qui se déroulent en même temps.*

*4. Mes pensées sont désorganisées et chaotiques.*

*5. Mes pensées sont constamment en mouvement.*

*6. J'ai une activité mentale incessante.*

*7. J'ai du mal à penser à quelque chose sans qu'une autre pensée n'entre dans mon esprit.*

*8. Je trouve que mes pensées sont distrayantes et m'empêchent de me concentrer sur ce que je fais.*

*9. J'ai du mal à ralentir mes pensées et à me concentrer sur une seule chose à la fois.*

*10. J'ai du mal à penser clairement, comme si mon esprit était dans le brouillard.*

*11. Je papillonne d'une pensée à l'autre.*

*12. Je ne peux me concentrer sur une seule chose qu'avec un effort considérable.*

# 2. Supplementary analyses and full model tables

## Supplementary materials for Section 3.1.1. Similarities of behavior between conditions

*Summary statistics of participant’s behavior*

|  | **nb. words/min.** | **WOA** | **proportion of repetitions** | **proportion of consecutive repetitions** |
| --- | --- | --- | --- | --- |
| Words | 26.38 (8.53) | 2.64 (1.16) | 0.05 (0.05) | 0 (0) |
| Words+OS | 25.11 (7.87) | 2.7 (0.92) | 0.04 (0.05) | 0 (0.01) |

**Table 1**. Descriptive statistics of word production. Format: mean (sd).

|  | **nb. on. seg./min.** | **on. seg. duration (sec.)** | **nb. off. seg./min.** | **prop. words in off. seg.** | **off. seg. size (nb. words)** |
| --- | --- | --- | --- | --- | --- |
| Words |  |  | 4.06 (1.73) | 0.93 (0.07) | 7.3 (3.57) |
| Words+OS | 4.11 (2.15) | 17.47 (7.82) | 3.66 (1.41) | 0.97 (0.05) | 7.4 (2.93) |
| InnerWords+OS | 4.62 (3.04) | 15.91 (7.56) |  |  |  |
| InnerThought+OS | 3.83 (3.01) | 20.33 (10.16) |  |  |  |

**Table 2**. Descriptive statistics of thought segmentation. Format: mean (sd). on. seg. = online segments; off. seg. = offline segments.

*Thought segmentation correlates across conditions*

We compared thought segmentation across conditions on the basis on the mean number of segments per minute to make the online and offline segmentations comparable.

|  | Words+OS-off | | Words+OS-on | | InnerWords+OS | | InnerThought+OS | |
| --- | --- | --- | --- | --- | --- | --- | --- | --- |
|  | *rho* | *p* | *rho* | *p* | *rho* | *p* | *rho* | *p* |
| Words | 0.82 | **< .001***** | 0.63 | **< .001***** | 0.49 | **< .001***** | 0.26 | .05 |
| Words+OS-off |  |  | 0.78 | **< .001***** | 0.59 | **< .001***** | 0.34 | **.01**** |
| Words+OS-on |  |  |  |  | 0.8 | **< .001***** | 0.54 | **< .001***** |
| InnerWords+OS |  |  |  |  |  |  | 0.67 | **< .001***** |

**Table 3.** Correlations between mean normalized number of thought segments. Words / WordsOS-off: offline segmentation; WordsOS-on / InnerWordsOS / InnerThoughtOS: online segmentation.

*Correlations between questionnaire scores and mean TSDs and segment sizes*

|  | ASRS score | | MEWS score | |
| --- | --- | --- | --- | --- |
|  | ***rho*** | ***p*** | ***rho*** | ***p*** |
| Words+OS | 0.06 | .66 | -0.04 | .75 |
| InnerWords+OS | -0.25 | .06 | -0.22 | .08 |
| InnerThought+OS | -0.20 | .13 | -0.33 | **.01*** |

**Table 4.** Correlations between questionnaire scores and mean duration of online segments (in seconds)

|  | ASRS score | | MEWS score | |
| --- | --- | --- | --- | --- |
|  | ***rho*** | ***p*** | ***rho*** | ***p*** |
| Words | -0.09 | .48 | -0.14 | .3 |
| WordsOS | -0.08 | .57 | -0.07 | .61 |

**Table 5.** Correlations between questionnaire scores and mean size of offline (retrospective) segments (in number of words)

We did not observe the same effect of smaller and more numerous offline segments for participants higher on the ASRS scale as in Kérébel et al. (2024). However, there are reasons to believe that in the present project the offline segmentation is less meaningful because of the presence of the online segmentation task in some conditions. For example, in the current dataset the proportion of words left outside of any segment was very low (3% to 7%, compared to 16% in Kérébel et al.’s data), suggesting that the online segmentation task—that imposes to put only punctual boundaries between segments—influenced the offline segmentation even in the *Words* condition where it was not performed.

## Supplementary materials for Section 3.1.2. Keypresses reflect thought transitions

*Supp. analysis 1: Online and Offline segmentations align*

For each keypress, we compute its distance to the closest offline cluster starting word. We averaged these distances by series and participant to obtain a divergence score (mean = 1.66, sd = 0.71). In comparison, the mean half size of an offline cluster was 3.7 (+/- 1.4) words. We turned to permutations to estimate the chance level in each series. We used two permutation methods: 1) randomly shuffling the position of the keypresses within each series, 2) randomly shuffling the position of the keypresses while keeping the same distribution of inter-keypress distances within each series. Each permutation yielded a noise divergence score. We ran 1000 permutations^[1]^ and found mean noise divergence scores of 2.65 (+/- 1.08) and 2.55 (+/- 1.01) for the methods 1) and 2) respectively. For all but two participants, the observed divergence scores were smaller than the noise divergence score, with a mean difference of 0.99 (+/- 0.96) and 0.89 (+/- 0.83) respectively. Note that not all divergences are necessarily bad: a keypress could fall in the middle of a segment but yet separate into two coherent sub-segments.

*Model tables*

| log(onSt)~nb_words + log(offSt) + (1 + log(offSt) \| subject_id) , family: gaussian | | | | | |
| --- | --- | --- | --- | --- | --- |
|  | **Estimate** | **Std. Error** | **t value** | **Pr(>\|t\|)** | **95% CI** |
| (Intercept) | 0.42 | 0.2 | 2.14 | **.038*** | [0.04; 0.8] |
| nb_words | 0.00 | 0.0 | 0.65 | .52 | [0; 0.01] |
| log(offSt) | 0.82 | 0.1 | 7.96 | **< .001***** | [0.62; 1.03] |
| Marginal r2: 0.49; Conditional r2: 0.76 | | | | | |

**Table 6.** Model coefficients. Words+OS condition. onSt = online segment, offSt = offline segment. The numbers of keypresses and offline segments were log-transformed in order to fulfill model assumptions. Data points above 120 sec. in the trial were removed in order to avoid the confound of trial duration.

| log(onSt)~1 + (1 + log(offSt) \| subject_id) , family: gaussian | | | | | |
| --- | --- | --- | --- | --- | --- |
|  | **Estimate** | **Std. Error** | **t value** | **Pr(>\|t\|)** | **95% CI** |
| (Intercept) | 2.17 | 0.04 | 54.72 | **< .001***** | [2.09; 2.25] |
| Marginal r2: 0; Conditional r2: 0.78 | | | | | |

**Table 7.** Null model coefficients. Words+OS condition. onSt = online segment, offSt = offline segment. The numbers of keypresses and offline segments were log-transformed in order to fulfill model assumptions. Data points above 120 sec. in the trial were removed in order to avoid the confound of trial duration.

| KP~consDist + WOA + Onset_s + (1 + WOA \| subject_id/series_nb) , family: binomial | | | | | | |
| --- | --- | --- | --- | --- | --- | --- |
|  | **Estimate** | **Std. Error** | **z value** | **Pr(>\|z\|)** | **95% CI** | **OR** |
| (Intercept) | -3.30 | 0.26 | -12.92 | **< .001***** | [-3.8; -2.8] | 0.04 |
| consDist | 0.42 | 0.52 | 0.82 | .41 | [-0.59; 1.43] | 1.52 |
| WOA | 0.51 | 0.05 | 9.32 | **< .001***** | [0.4; 0.62] | 1.67 |
| Onset_s | 0.65 | 0.61 | 1.06 | .29 | [-0.54; 1.84] | 1.92 |

**Table 8.** Model coefficients. Words+OS condition. KP = key press.

| KP~1 + (1 + WOA \| subject_id/series_nb) , family: binomial | | | | | | |
| --- | --- | --- | --- | --- | --- | --- |
|  | Estimate | Std. Error | z value | Pr(>\|z\|) | 95% CI | OR |
| (Intercept) | -1.74 | 0.13 | -13.82 | **< .001***** | [-1.99; -1.49] | 0.18 |

**Table 9.** Null model coefficients. Words+OS condition. KP = key press.

**Figure 1.** Mean consecutive semantic distances in the *Words+OS* condition. “post KP”: words immediately following a key press, “other”: other words.

## Supplementary materials for Section 3.1.3: Thought transitions are driven both by semantics and elapsed time

| preKP~nSec + lastKPDist + consDist + (1 + lastKPDist \| subject_id:series_nb) , family: binomial | | | | | | |
| --- | --- | --- | --- | --- | --- | --- |
|  | **Estimate** | **Std. Error** | **z value** | **Pr(>\|z\|)** | **95% CI** | **OR** |
| (Intercept) | -4.11 | 0.33 | -12.55 | **< .001***** | [-4.75; -3.46] | 0.02 |
| nSec | 0.02 | 0.00 | 6.59 | **< .001***** | [0.02; 0.03] | 1.02 |
| lastKPDist | 2.86 | 0.76 | 3.78 | **< .001***** | [1.38; 4.35] | 17.46 |
| consDist | 2.66 | 0.55 | 4.87 | **< .001***** | [1.59; 3.73] | 14.30 |

**Table 10**. Model coefficients of the best model considered on section *3.1.3*. Words+OS condition.

| preKP~1 + (1 + lastKPDist \| subject_id:series_nb) , family: binomial | | | | | | |
| --- | --- | --- | --- | --- | --- | --- |
|  | **Estimate** | **Std. Error** | **z value** | **Pr(>\|z\|)** | **95% CI** | **OR** |
| (Intercept) | -1.66 | 0.05 | -35.33 | **< .001***** | [-1.75; -1.57] | 0.19 |

**Table 11**. Coefficients of the null model corresponding to the best model considered on section *3.1.3*. Words+OS condition.

| **Formula** | **BIC** | **AIC** | **BF** |
| --- | --- | --- | --- |
| preKP ~ nSec + lastKPDist + consDist | 8857.34 | 8821.41 | x |
| preKP ~ nSec + consDist | 8868.83 | 8840.08 | < 0.1 |
| preKP ~ cumConsDist + lastKPDist + consDist | 8872.31 | 8836.38 | < 0.1 |
| preKP ~ nSec + lastKPDist | 8873.14 | 8844.39 | < 0.1 |
| preKP ~ cumConsDist + nSec + consDist | 8878.00 | 8842.06 | < 0.1 |
| preKP ~ nSec + lastKPDist + WOA | 8881.11 | 8845.18 | < 0.1 |
| preKP ~ cumConsDist + lastKPDist + WOA | 8888.39 | 8852.45 | < 0.1 |
| preKP ~ lastKPDist + consDist | 8891.02 | 8862.27 | < 0.1 |
| preKP ~ cumConsDist + nSec + WOA | 8925.99 | 8890.05 | < 0.1 |

**Table 12.** Comparison of the test model (top row) against alternative models including temporal predictors. BIC: Bayesian Information Criterion, AIC: Akaike Information Criterion, BF: (approximated) Bayes Factor representing the evidence in favor of the alternative models.

*Supp. analysis 2: Progressive or transient increases in semantic distance prior to keypresses?*

We wanted to assess whether the increase of consecutive semantic distance (consDist) and semantic distance to the last transition word (lastKPDist) before keypresses were progressive or transient. For each of the two variables, we started by running a linear model predicting its value on the 7 lasts words of each segment (median size of thought segments), based on the position of the words relative to the next keypress. We observed positive effects of the position for both variables (consDist: est. = 0.02, p < 0.001; lastKPDist: est. = 0.02, p < 0.001 see Supp. Tables 13 and 15). We then tested whether these effects were driven by the word immediately preceding keypresses by rerunning the same model without the last word. The effect remained for the lastKPDist (est. = 0.01, p = 0.005), but was not significant anymore for the consDist (p = 0.08), suggesting a step-like increase prior to keypresses.

Visual inspection of the data (Supp. Fig. 2) suggested a steeper increase for the last word before the keypress for both variables. We tested it by grouping the word positions into pairs (-4 and -3, -2 and -1) and looking at the interaction between the pair and the position inside the pair (1 or 2). We observed a pair:position-in-pair interaction for both variables (consDist: est. = 0.04, p < 0.001; lastKPDist: est. = 0.03, p < 0.001, see Supp. Tables 17 and 19), confirming a steeper increase closer to the transition, and a positive effect of the pair rank for lastKPDist only (est. = 0.03, p = 0.04). For the analyses reported in this paragraph we removed outlier values below mean+2*sd to cope with models’ assumptions.

**Figure 2.** Semantic distances based on the position relative to the last word of the segment. Left: Semantic distance to the first word of the thought segment (lastKPDist). Right: Consecutive semantic distance (consDist).

| I(lastKPDist * 10) ~ position_pre + (1+ position_pre \| subject_id:series_nb:last_KP_index) | | | | | |
| --- | --- | --- | --- | --- | --- |
|  | **Estimate** | **Std. Error** | **t value** | **Pr(>\|t\|)** | **95% CI** |
| (Intercept) | 4.18 | 0.01 | 414.23 | **< .001***** | [4.16; 4.2] |
| position_pre | 0.02 | 0.00 | 7.21 | **< .001***** | [0.01; 0.02] |
| Marginal r2: 0.01; Conditional r2: 0.3 | | | | | |

**Table 13.** Model coefficients. Words+OS condition, words within 7 words of a keypress. position_pre: position relative to the next keypress (-7, -6, -5, -4, -3, -2, or -1).

| I(lastKPDist * 10)~1 + (1 + position_pre \| subject_id:series_nb:last_KP_index) | | | | | |
| --- | --- | --- | --- | --- | --- |
|  | **Estimate** | **Std. Error** | **t value** | **Pr(>\|t\|)** | **95% CI** |
| (Intercept) | 4.13 | 0.01 | 609.45 | **< .001***** | [4.11; 4.14] |
| Marginal r2: 0; Conditional r2: 0.3 | | | | | |

**Table 14.** Null model coefficients. Words+OS condition, words within 7 words of a keypress. position_pre: position relative to the next keypress (-7, -6, -5, -4, -3, -2, or -1).

| I(consDist * 10)~position_pre + (1 + position_pre \| subject_id:series_nb:last_KP_index) | | | | | |
| --- | --- | --- | --- | --- | --- |
|  | Estimate | Std. Error | t value | Pr(>\|t\|) | 95% CI |
| (Intercept) | 3.98 | 0.01 | 327.91 | **< .001***** | [3.95; 4] |
| position_pre | 0.02 | 0.00 | 5.45 | **< .001***** | [0.01; 0.02] |
| Marginal r2: 0; Conditional r2: 0.16 | | | | | |

**Table 15.** Model coefficients. Words+OS condition, words within 7 words of a keypress. position_pre: position relative to the next keypress (-7, -6, -5, -4, -3, -2, or -1).

| I(consDist * 10)~1 + (1 + position_pre \| subject_id:series_nb:last_KP_index) | | | | | |
| --- | --- | --- | --- | --- | --- |
|  | Estimate | Std. Error | t value | Pr(>\|t\|) | 95% CI |
| (Intercept) | 3.92 | 0.01 | 555.87 | **< .001***** | [3.91; 3.94] |
| Marginal r2: 0; Conditional r2: 0.16 | | | | | |

**Table 16.** Null model coefficients. Words+OS condition, words within 7 words of a keypress. position_pre: position relative to the next keypress (-7, -6, -5, -4, -3, -2, or -1).

| I(lastKPDist * 10)~pair + posInPair:pair + (1 + pair \| subject_id:series_nb:last_KP_index) | | | | | |
| --- | --- | --- | --- | --- | --- |
|  | Estimate | Std. Error | t value | Pr(>\|t\|) | 95% CI |
| (Intercept) | 4.01 | 0.02 | 221.10 | **< .001***** | [3.98; 4.05] |
| pair | 0.03 | 0.01 | 2.05 | **.04*** | [0; 0.05] |
| pair:posInPair | 0.03 | 0.01 | 5.67 | **< .001***** | [0.02; 0.04] |
| Marginal r2: 0.01; Conditional r2: 0.34 | | | | | |

**Table 17.** Model coefficients. Words+OS condition. pair: 1 = positions -4 and -3, 2 = positions -2 and -1, posInPair: position inside the pair (1 or 2).

| I(lastKPDist * 10)~1 + (1 + pair \| subject_id:series_nb:last_KP_index) | | | | | |
| --- | --- | --- | --- | --- | --- |
|  | Estimate | Std. Error | t value | Pr(>\|t\|) | 95% CI |
| (Intercept) | 4.13 | 0.01 | 566.99 | **< .001***** | [4.12; 4.15] |
| Marginal r2: 0; Conditional r2: 0.33 | | | | | |

**Table 18.** Null model coefficients. Words+OS condition. pair: 1 = positions -4 and -3, 2 = positions -2 and -1, posInPair: position inside the pair (1 or 2).

| I(consDist * 10)~pair + posInPair:pair + (1 + pair + posInPair:pair \| subject_id:series_nb:last_KP_index) | | | | | |
| --- | --- | --- | --- | --- | --- |
|  | Estimate | Std. Error | t value | Pr(>\|t\|) | 95% CI |
| (Intercept) | 3.85 | 0.02 | 174.55 | **< .001***** | [3.81; 3.89] |
| pair | 0.00 | 0.02 | -0.10 | .92 | [-0.03; 0.03] |
| pair:posInPair | 0.04 | 0.01 | 4.74 | **< .001***** | [0.02; 0.05] |
| Marginal r2: 0.01; Conditional r2: 0.18 | | | | | |

**Table 19.** Model coefficients. Words+OS condition. pair: 1 = positions -4 and -3, 2 = positions -2 and -1, posInPair: position inside the pair (1 or 2). The corresponding null model is not reported here as it resulted in a singular fit.

## Supplementary materials for Section *3.2. Thought segment durations follow a Wald distribution*

*Model space*

| **Model** | **Distribution of the TSD** | **Probability density function** | **Parameters meaning** |
| --- | --- | --- | --- |
| *Constant probability* | Exponential |  | λ: rate of the Poisson process |
| *Inner metronome* | (truncated) Gaussian |  | μ: mean  σ: standard deviation |
| *Accumulator* | Wald |  | γ: accumulation rate  α: accumulation threshold |
| *Accumulator + non-accumulation time* | shifted-Wald |  | γ: accumulation rate  α: accumulation threshold  θ: non-accumulation time |

**Table 20.** Model space. TSD: thought segment durations

*Fitting procedure*

The models were fitted using the fitdist function of the fitdistr R package (Delignette-Muller & Dutang, 2015). We used the following arguments:

- mod1 (exp): starting value: λ=1/mean(val); method="mle"
- mod2 (norm): starting values: μ0=mean(val), σ0=sd(val); method="mle"
- mod3a (Wald): starting values: γ0=sqrt(mean(val-min(val))/var(val-mean(val))), α0=mean(val-min(val))*γ0; method="mle"; lower bounds: 0
- mod3b (shifted-Wald): starting values: theta0=0.00001, γ0=sqrt(mean(val-min(val))/var(val-mean(val))), α0=mean(val-min(val))*γ0; method="mle"; lower bounds: 0

For each model, we estimated the best-fitted set of parameters so as to minimize the difference between the observed distribution of online segments and the probability density function of the model.

*Model fits on the Online Control Task*

| *Online Segmentation Control* | |
| --- | --- |
| **model** | **nb. best** |
| *W* | 54 |
| *norm* | 4 |
| *exp* | 1 |
| *sW* | 1 |

**Table 21.** Results of model comparisons. Models fitted on thought segment durations (TSD) on data from the Online Segmentation Control condition (OSC).

*Parameter recovery*

In order to assess the robustness of our modeling results, we simulated data and used our fitting procedure to recover the simulation parameters. We did it for the ‘weakest’ situation, i.e. the condition and measure on which we had the lowest data point: the TSD in the *InnerThought+OS* condition.

For each simulation we used the following procedure. First, a pair of parameters (alpha and gamma) was drawn from ‘reasonable’ distributions (see below). Second, for the three trial durations of the task (120, 150, and 180 sec.), we sampled values from the corresponding Wald distribution until the sum exceeded trial duration. Third, the values from the three trials were pooled in a single distribution, on which we fitted a Wald distribution. This procedure was repeated 500 times. We removed 8 simulations on which fitting failed to converge, and 21 on which the resulting number of samples was smaller than the smallest number of keypresses in our data.

We observed strong correlations between simulation parameters and fitted parameters (alpha: r = 0.83, p < .001; gamma: r = 0.73, p < .001, see Supp. Fig. 4), meaning we were able to recover the simulation parameters consistently.

The “reasonable” simulation parameters were chosen by fitting two gaussian distributions on the parameters fitted on participants data (one per parameter), and then drawing values from these distributions for each simulation (Danwitz et al., 2022).

Note that doing simulations this way is closer to the real data structure than just taking a given number of samples: this allowed us to render the fact that the participants with a high number of keypresses necessarily exhibited small TSDs.

**Figure 3.** Correlation between simulation parameters and recovered parameters. Color: number of samples in the simulation. Black solid line: identity diagonal. Gray line: outlier detection threshold used on the real data. Black dashed line: mean+2*SD threshold on the recovered parameters.

*Model recovery*

We then checked whether we were able to correctly differentiate between models with our sample size. For each of the three candidate models, we simulated 500 participants with ‘reasonable’ parameters (see above). We then fitted the three models on each simulated participant and we compared the quality of each fit using the BIC. For the three distributions we achieved nearly perfect model recovery (proportions of successful model recovery: Wald: 0.99; Exponential: 0.99; Gaussian: 0.85).

Note that this time we simulated data for the TSD on three conditions (i.e. 9 trials) since in the main analysis we compared the models based on the data of the *Words+OS*, *InnerWords+OS*, and *InnerThought+OS* conditions.

*Outlier fits*

As a way to identify bad parameter estimation, we excluded the fits whose parameters were two standard deviations above the group average for the correlation analyses (Figure 4). We found the same pattern of results when running the analyses on all the participants (Table 18).

**Figure 4.** distribution of the Wald model parameters for thought segment durations (TSD), and word onset asynchrony (WOA), fitted on the data from *all* conditions, *Thought+KP* condition only, and online segmentation control task (*OnSC*). Black dashed line: mean, grey dashed line: mean+2*sd, grey dotted line: mean+3*sd. Colors: each color represents a participant (consistent across all figures).

*Supp. analysis 3: Modelling of word onset asynchrony (WOA)*

In our task, the WOAs are somewhat close to reaction times, which are often modelled using accumulators. Hence, we also ran our model fitting procedure on the WOAs, both for completeness and as another quality check.

Results of the model comparison are reported on Table 19. We tested the correlation between the accumulation rates resulting from the fits of the Wald distribution on the distribution of WOAs and of TSDs at the individual level. Interestingly, there was a positive correlation between accumulation rates (r = 0.48, p < 0.001, BF = 124.45), but not between thresholds (r = 0.218, p = 0.12, BF = 0.94).

| **model** | **nb. best** | **BIC pooled data** |
| --- | --- | --- |
| *W* | 36 | 68576.97 |
| *sW* | 21 | 68586.99 |
| *norm* | 2 | 81839.49 |
| *exp* | 0 | 87430.48 |

**Table 22**. Results of the model comparison. Models fitted on word onset asynchrony (WOAs). *Words* and *Words+OS* conditions pooled. For each line, “nb. best” indicates the number of participants whose data was best fitted by the corresponding model, and “BIC pooled data” indicates the Bayesian Information Criterion when fitting the model on the pooled data of all subjects.

## Supplementary materials on Section 3.3. Accumulation thresholds correlate with inattention and tendency toward disorganized thought

|  | **ASRS score** | | **MEWS score** | |
| --- | --- | --- | --- | --- |
|  | ***r*** | ***p*** | ***r*** | ***p*** |
| α all | -0.41 | **.002**** | -0.31 | **.018*** |
| α control | -0.16 | .25 | -0.06 | .66 |
| α Words+OS | -0.27 | **.045*** | -0.16 | .24 |
| α InnerWords+OS | -0.48 | **< .001***** | -0.29 | **.031*** |
| α InnerThought+OS | -0.32 | **.019*** | -0.35 | **.011*** |
| γ all | -0.20 | .12 | 0.01 | .95 |
| γ control | -0.04 | .79 | 0.15 | .27 |
| γ Words+OS | -0.20 | .13 | -0.10 | .49 |
| γ InnerWords+OS | -0.15 | .27 | 0.08 | .53 |
| γ InnerThought+OS | -0.05 | .7 | 0.13 | .34 |

**Table 23.** Correlation between questionnaire scores and accumulation parameters fitted on each condition.

|  | **ASRS score** | | **MEWS score** | |
| --- | --- | --- | --- | --- |
|  | ***r*** | ***p*** | ***r*** | ***p*** |
| α all | -0.40 | **.001**** | -0.20 | .13 |
| α control | -0.10 | .47 | -0.04 | .75 |
| α InnerThought+OS | -0.29 | **.029*** | -0.32 | **.014*** |
| γ all | -0.12 | .34 | 0.10 | .46 |
| γ control | -0.04 | .75 | 0.11 | .41 |
| γ InnerThought+OS | -0.03 | .8 | 0.12 | .36 |

**Table 24.** correlations between model parameters and questionnaires without excluding the outlier fits. Note that we take the outlier parameter values to be a sign of poor fit rather than extreme behavior. r: Pearson’s coefficient.

## Other supplementary analyses

*Supp. analysis 4: Correlation between questionnaire scores*

There was a positive correlation between the MEWS and ASRS questionnaires (r = 0.56, p < 0.001).

**Figure 5.** Correlation between ASRS score and MEWS score.

*Supp. analysis 5: Segmentation events do not depend on position in time*

We checked if there was any systematic bias regarding when participants indicated transitions within the series. In order to do that, we split the trials in chunks of 30 seconds and tested whether there was an effect of the bin on the mean number of recorded keypresses. A within-subject ANOVA run on the first 120 seconds of each trial (all conditions) revealed no effect of the time bin (F(2.2,122) = 1.19, p = 0.31, BF = 0.11).

*Supp. analysis 6: Autocorrelation of TSDs*

We assessed the auto-correlation of thought segments durations at lag=1 using a linear mixed effect model. We found a positive effect of the duration of TSD n-1 on TSD n (see Table 13 below).

| log(onlineSeg_duration)~log(TSD_Nm1) + (1 + log(TSD_Nm1) \| subject_id:series_nb) | | | | | |
| --- | --- | --- | --- | --- | --- |
|  | **Estimate** | **Std. Error** | **t value** | **Pr(>\|t\|)** | **95% CI** |
| (Intercept) | 2.23 | 0.05 | 43.69 | **< .001***** | [2.13; 2.33] |
| log(TSD_Nm1) | 0.11 | 0.02 | 6.31 | **< .001***** | [0.08; 0.15] |
| Marginal r2: 0.02; Conditional r2: 0.37 | | | | | |

**Table 25.** Model coefficients. Conditions Words+OS, InnerWords+OS and InnerThoughts+OS pooled.

| log(onlineSeg_duration)~1 + (1 + log(TSD_Nm1) \| subject_id:series_nb) | | | | | |
| --- | --- | --- | --- | --- | --- |
|  | Estimate | Std. Error | t value | Pr(>\|t\|) | 95% CI |
| (Intercept) | 2.55 | 0.02 | 115.76 | **< .001***** | [2.51; 2.6] |
| Marginal r2: 0; Conditional r2: 0.41 | | | | | |

**Table 26.** Null model coefficients. Conditions Words+OS, InnerWords+OS and InnerThoughts+OS pooled.

**References**

Bonin, P., Méot, A., Ferrand, L., & Bugaïska, A. (2013). Normes d’associations verbales pour 520 mots concrets et étude de leurs relations avec d’autres variables psycholinguistiques: *L’Année psychologique*, *Vol. 113*(1), 63‑92. https://doi.org/10.3917/anpsy.131.0063

Caci, H. M., Morin, A. J. S., & Tran, A. (2014). Prevalence and correlates of attention deficit hyperactivity disorder in adults from a French community sample. *The Journal of Nervous and Mental Disease*, *202*(4), 324‑332. https://doi.org/10.1097/NMD.0000000000000126

Danwitz, L., Mathar, D., Smith, E., Tuzsus, D., & Peters, J. (2022). Parameter and Model Recovery of Reinforcement Learning Models for Restless Bandit Problems. *Computational Brain & Behavior*, *5*(4), 547‑563. https://doi.org/10.1007/s42113-022-00139-0

Delignette-Muller, M. L., & Dutang, C. (2015). **fitdistrplus** : An *R* Package for Fitting Distributions. *Journal of Statistical Software*, *64*(4). https://doi.org/10.18637/jss.v064.i04

Kérébel, A., Caille, J.-A., & Sackur, J. (2024). Dynamics of spontaneous thoughts : Exploration, attentional profile and the segmentation of the stream of thoughts. *Consciousness and Cognition*, *124*, 103735. https://doi.org/10.1016/j.concog.2024.103735

Mowlem, F. D., Skirrow, C., Reid, P., Maltezos, S., Nijjar, S. K., Merwood, A., Barker, E., Cooper, R., Kuntsi, J., & Asherson, P. (2019). Validation of the Mind Excessively Wandering Scale and the Relationship of Mind Wandering to Impairment in Adult ADHD. *Journal of Attention Disorders*, *23*(6), 624‑634. https://doi.org/10.1177/1087054716651927

New, B., Pallier, C., Brysbaert, M., & Ferrand, L. (2004). Lexique 2 : A new French lexical database. *Behavior Research Methods, Instruments, & Computers*, *36*(3), 516‑524. https://doi.org/10.3758/BF03195598

1. Note that depending on the number of words or clusters in a given trial, the total number of possible permutations was often below 1000.
